# SEROTONIN DEFICIENCY FROM CONSTITUTIVE SKN-1 ACTIVATION DRIVES PATHOGEN APATHY

**DOI:** 10.1093/geroni/igae098.3711

**Published:** 2024-12-31

**Authors:** Brandy Weathers, Tripti Nair, Sean Curran, James Nhan, Nicole Sthur

**Affiliations:** University of Southern California, Los Angeles, California, United States; University of Southern California, Los Angeles, California, United States; University of Southern California, Los Angeles, California, United States; University of Southern California, Los Angeles, California, United States; University of Southern California, Los Angeles, California, United States

## Abstract

When an organism encounters a pathogen, the host innate immune system activates to defend against pathogen colonization and toxic xenobiotics produced. In humans, nuclear factor erythroid 2-related factor (Nrf2) is a critical regulator in innate immune responses under stress; it regulates gene expression in response to the oxidative stress induced by pathogens and is activated within innate immunity cells in response to pathogenic stress. Caenorhabditis elegans activate defense responses to ensure survival upon exposure to pathogens, including activation of the cytoprotective transcription factor SKN-1, a homolog of human Nrf2. SKN-1 directs homeostasis against cellular stress, including pathogenic stress. Exposure to Pseudomonas aeruginosa (PA14) revealed that animals with constitutive activation of SKN-1 develop an apathy-like behavior towards the pathogenic bacteria. Though wild-type C. elegans adapt to avoid pathogens, animals with constitutive SKN-1 activation choose not to leave while continuing to feed on the pathogen. This behavior is mediated by tissue-specific actions of SKN-1 that initiate cell non-autonomous outcomes for pathogen avoidance, survival, and metabolic adaptation. Remarkably, although animals with SKN-1 activation are initially apathetic to leaving behaviors associated with PA14 exposure, treatment with exogenous serotonin significantly reduces this phenotype. SKN-1 activation in neurons and intestinal tissues triggers a distinct transcriptional defect in serotonin signaling from both neurons and non-neuronal tissues, and a significant reduction of serotonin production. This altered signaling is the driving force behind the pathogen apathy behavior observed. Taken together, this work defines the role(s) of the SKN-1-serotonin-immunomodulatory pathway in organismal health and aging and in pathogen defenses.

